# Sentinel lymph node mapping by indocyanin green fluorescence imaging in oropharyngeal cancer - preliminary experience

**DOI:** 10.1186/1758-3284-2-31

**Published:** 2010-10-30

**Authors:** Marius G Bredell

**Affiliations:** 1 Department of Craniomaxillofacial Surgery, University Hospital of Zürich, Zürich, Switzerland

## Abstract

**Background:**

Sentinel lymph node (SLN) detection and biopsy is gaining popularity in the treatment of Head and Neck cancer. Various methods in this regard have been described, each with their respective advantages and disadvantages. The aim of this paper was to evaluate the potential application of Indocyanin Green (ICG) in the mapping and detection of sentinel lymph nodes (SLN) in cancers of the head and neck.

**Methods:**

Patients with oropharyngeal cancer and N0 neck who were scheduled for primary tumor ablation as well as a neck dissection were selected. One milliliter of Indocyanin green was injected around the tumor and the sentinel node detection was performed by aiming the infra red video camera on the cervical area. When no detection was possible transcutaneously, a cervical incision was made, a sub-platysmal flap raised and further detection was done to visualize the fluorescing lymph nodes.

**Results:**

Detection of cervical SLN was only possible when 5 mm or less tissue covered the sentinel lymph node. Accurate and clear detection of the lymph drainage pattern and SLN was possible. There is some uptake in other tissues such as the submandibular gland which is easily distinguishable from lymphatic tissue.

**Conclusion:**

Indocyanin green fluorescence is a potential valuable potential tool in the detection of SLN in patients with oropharyngeal cancer which warrants further investigation.

## Background

It is well known that poorer prognosis prevails in head and neck cancer patients that develop metastatic lymph nodes and even more so if there is evidence of extra capsular spread. This is one of the reasons why elective neck dissections are frequently performed even in patients with N0 necks [1].

There are no clear and accurate guidelines regarding the decision to perform a neck dissection concurrent to the ablation of the primary tumor in the N0 neck. Clinical staging of the neck by manual examination is notoriously inaccurate, especially in patients with more voluminous necks. Radiological staging with either CT or MRI has improved the sensitivity for identifying nodal metastatic disease and has contributed to the upstaging of necks in a significant number of patients, resulting in a more accurate selection of patients requiring a neck dissection. With size alone as one of the more important criteria in this regard it has been shown that an important group of patients at risk for occult lymphatic metastasis may be missed [2,3].

The potential benefit of CT-PET in the pre operative evaluation of patients with a negative CT or MRI is still controversial and probably plays a bigger role in detecting second primary tumors and follow up [4,5].

Ultrasound with simultaneous fine needle aspiration cytology is probably the most accurate method to date in staging the neck, but requires skill and solid ultrasound and cytology knowledge to reach good levels of accuracy [6].

Currently there is thus no accurate way of determining the presence of occult lymphatic metastasis for cancers of the head and neck. It is clear that the gold standard for detecting involved lymph nodes is accurate pathological analysis of the nodes. This is traditionally done in a retrospective manner by sending the neck dissection specimen for analysis. In the normal pathological processing there is a possibility to miss micro metastases as the specimens are not as accurately examined as with a sentinel node protocol. In such a protocol stepwise serial sectioning as well as immunohistochemistry will be able to find occult microscopic deposits that would be missed with routine pathology examinations [7].

Many factors determine the propensity of a tumor to disseminate its cells to spread via the anatomical lymphatic system. Tumor cell motility as well as lymphatic vessel density is factors that may contribute to the enhanced spread along lymphatic pathways [8].

The principle of sentinel lymph node (SLN) identification and biopsy is to identify the first station of drainage of a particular anatomical area in which the tumor lies [9,10].

SLN detection was first used in other anatomical areas like the breast and soon skin cancer as well as gastric cancers followed. In many centers SLN detection has become standard clinical practice.

Benefits for the patients are the more selective removal of the first drainage lymph nodes from a particular anatomical site with lower morbidity and by closer histological examination of the nodes a higher detection rate of micro metastasis is possible.

The anatomical basis of head and neck cancer nodal drainage has been well established and is in use on a daily basis [11]. On this basis the type as well extent of the neck dissection is determined [12]. For a long time the head and neck area has escaped the concept of sentinel neck dissection due to the conceived variability in the lymph drainage from the oropharyngeal area. In recent years this concept has made way for increasing interest in the potential benefits in patients with oropharyngeal cancer.

SLN identification and biopsy has increased its role in detection of the first nodal drainage in an attempt to either limit surgical damage, or to more accurately stage the patient by more accurate examination of the sentinel node or nodes. Koch et al (1998) was the first to describe the use of technesium-99 m (99T cm) labeled sulfur colloid in the quest for accurate sentinel node biopsy [13].

The use of lymphoscyntography with 99T cm colloid as well as a following SLN biopsy with possible concurrent blue dye injection can be regarded as the current gold standard with regards to identification of the SLN [14]. With this technique there is no direct visualization of the lymph drainage pattern, but only indirect visualization of the SLN with the gamma probe. Indocyanin Green (ICG) has been used extensively in the medical field since 1957 [15]. Initial uses were for blood flow measurements, but of late one of the mainstay uses is for retinal angiography, especially in the diagnosis of wet age related macular degeneration. As soon as ICG is infused intravenously, it rapidly binds to plasma proteins and thereby is confined to the vascular space. ICG is removed exclusively by the liver at the rate of 18-24% per minute so the elimination of ICG follows an exponential curve with a half-life of ~150-180 s [16].

The use of ICG in the diagnosis of SLN in areas other than that of the head and neck region has well been described. Reports of its use in gastric, breast, lung as well as skin cancer are found in the literature with good success [17-22]

## Methods

A total of 8 Patients with oropharyngeal head and neck cancer and N0 neck status and where resection of the primary tumor as well as a neck dissection was planned were used to evaluate the use of ICG in the identification of the sentinel lymph node. Only unilateral elective neck dissections were performed according to the clinical indication. In no way was the treatment of the patients altered or influenced by the additional use of ICG or sentinel node detection. Patient's informed consent was obtained before surgery.

**Figure 1:**
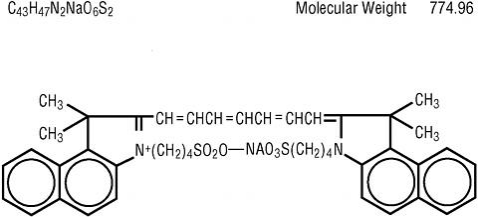
**Molecular structure of Indocyanin green**.

ICG (^®^ICG-Pulsion, Pulsion medical Systems AG, Germany)is supplied as a sterile, water-soluble powder dye (Figure 1) that is used clinically as a dilution indicator for studies involving the heart, liver, lungs, and circulation as well as for ophthalmic angiography.

ICG contains sodium iodide and should be used with caution in patients who have a history of allergy to iodides.

**Figure 2:**
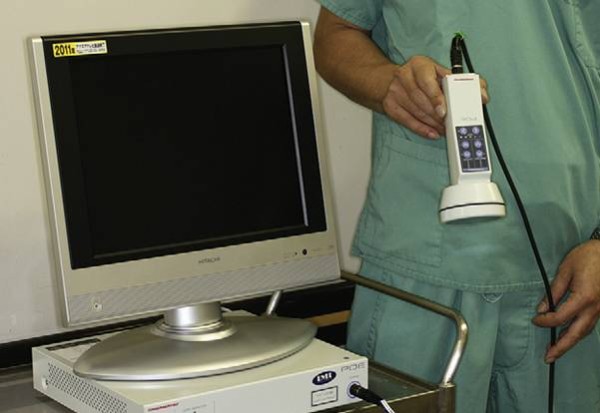
**Infra red camera system**.

All patients were treated under general anesthesia. Standard nasal or tracheotomy intubation was performed. A solution of ICG is made up by diluting 50 mg ICG powder with 5 ml aqueous water. The proposed mucosal resection margins are marked with superficial diathermy or CO2 laser points for the possibility of mucosal color changes after the ICG injection. Along the circumference of the tumor one milliliter of ICG solution was injected with at least five injection points. Special care has to be taken to prevent spill around the tumor area as well as contamination on gloves that may be transferred to the skin that will result in inappropriate light reaction in unaffected areas and interpretation of the signal. The infra red video camera (^®^PDE-Photodynamic Eye, Hamamatsu Photonics Deutschland GmbH) was then directed to the cervical area within 3-5 minutes and an attempt was made to identify the sentinel lymph node. (Figure 2) There are three possibilities to adjust the camera to gain optimal visualization and identification of the sentinel node, namely brightness, infrared and contrast.

Due to poor initial experiences with the trans-cutaneous visualization a sub-platysmal flap was raised and the sternocleidomastoid muscle was retracted before the Infra red camera was directed to the area. Time from injection to identification was more than 30 minutesinitially, but brought down to 5 minutes or less in the latter cases where the lymphatic drainage pattern could be seen in real time on the video screen.

The lymph drainage pattern was noted and sentinel node or nodes were marked and sent separately for sentinel node pathological examination.

All the sentinel nodes were marked and sent to the pathologist for evaluation according to a sentinel node protocol. The planned neck dissection was then completed as planned.

## Results

**Figure 3:**
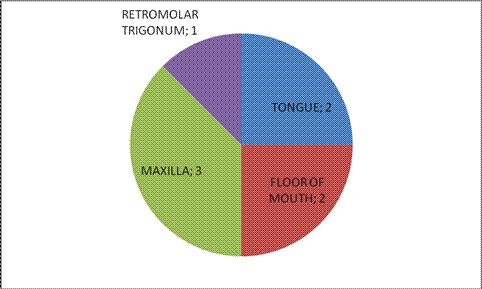
**Anatomical sites involved**.

In total 8 patients with oropharyngeal cancer (Figure 3) and N0 necks were subjected to the added investigation as described. In all cases the sentinel node or nodes could be identified and sent for separate, more intense pathological identification. The number of sentinel lymph nodes varied from one to 5 with an average of 3. In one patient with a maxillary tumor the sentinel node was positive with a micro metastasis present.

**Figure 4:**
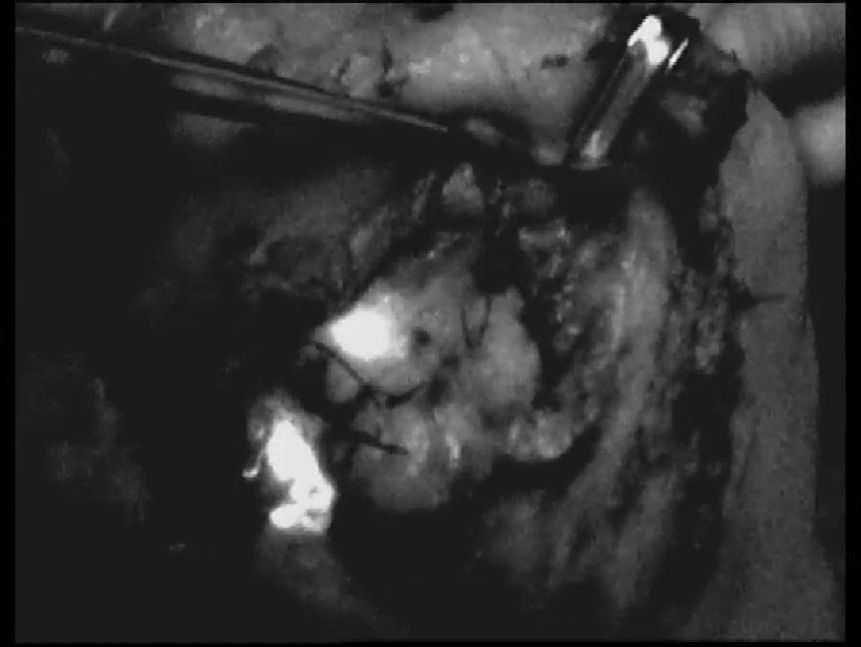
**Level IB**.

**Figure 5:**
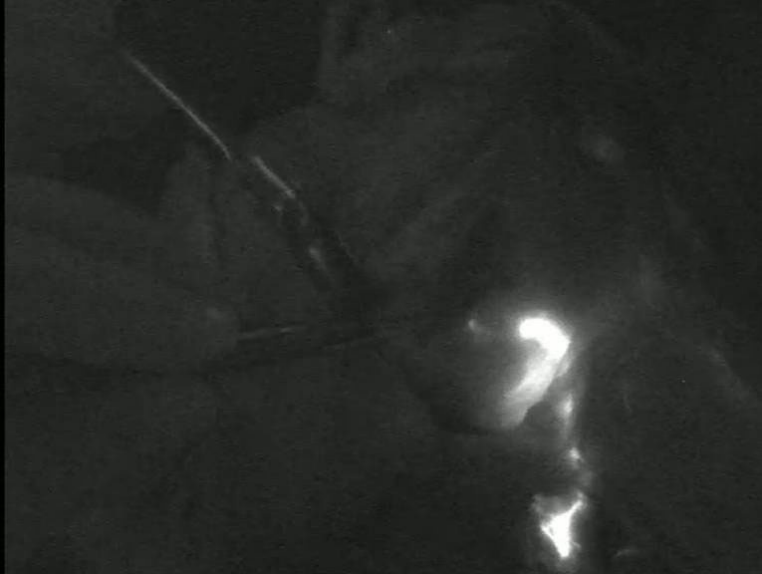
**Jugulodigastric lymph nodes with fluorescence**.

Identification of the SLN takes some getting used to as the node has to be exposed to some extent before the light reaction can be visualized through the infra red video camera. All cases where identification was attempted through the skin were unsuccessful. Thereafter the skin incision and exposure of the neck in a sub-platysma muscle level and exposure of the levels IA and IB with an attempt to identify the first station of lymph drainage followed. (Figure 4) In the next step the Sternocleidomastoid muscle was retracted and the Jugulodigastric lymph nodes were evaluated by the Infra red video image for possible sentinel nodes. (Figure 5) There was enough time (more than two hours) to complete the neck dissection and identify the SLN of the contralateral side in one case. It was clear that the longer the lag time from injection to visualization the more lymph nodes were identified. A subjective ideal time was later set at 5 minutes after injection.

**Figure 6:**
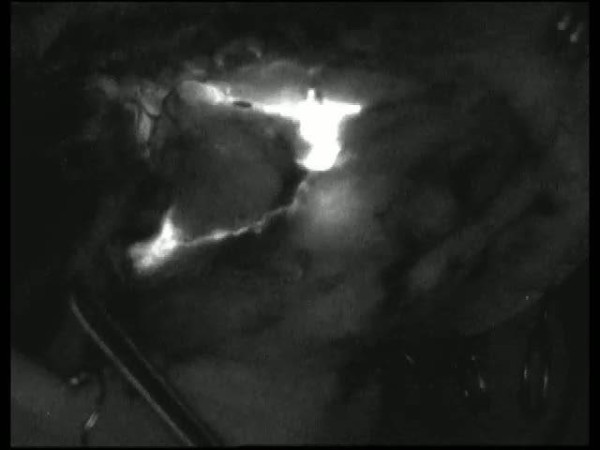
**Lymph drainage around the submandibular gland**.

In two cases the lymphatic drainage pattern around the submandibular gland could be identified and followed to the appropriate sentinel lymph node. (Figure 6) A further two cases demonstrated distinct ICG uptake in the submandibular gland that did not represent lymphatic tissue. As soon as more than 5 mm tissue covers the SLN it will probably hinder the identification of the fluorescent tissue.

There is a distinct learning curve involved in the use and finding the optimal balance between brightness, infra red and contrast settings with the infra red video camera.

Direct light like the operating light will interfere with the picture quality and visualization ability and may need to be dimmed or switched off in order to optimize the visualization of fluorescent SLN. Settings on the video camera may be set in such a way that there the surgical field and tissues are identifiable on the monitor.

## Discussion

The identification of sentinel lymph nodes in the head and neck area is still controversial although now more and more used in the clinically N0 neck situation. SLN biopsy is regarded valuable for two main reasons. One is the possible better identification of the anatomically first drainage lymph node in the drainage basin and should have the highest risk for being the first affected in the case of metastatic disease. Once the node or nodes are identified the node can be sent for frozen section and when positive a complete neck dissection is performed. If negative the patient received a procedure with lowered morbidity with a high chance of the neck being treated effectively and efficiently. Secondly the other alternative is to progress to a planned neck dissection and merely mark the SLN and request intensified pathological workup of the SLN that may lead to an upstaging of the neck of 34-40% [14,23,24].

Many methods and components have been described to be used in SLN identification. The first and simplest of these is methylene blue which can be used as a fluorescent component as well. Methylene blue can be combined with ^125^I as an easier intraoperative protocol with less radiation exposure [24].

Technesium 99 labelled sulfur colloid (^99 m^TcSC) alone or in combination with methylene blue is probably the most widely used as it allows one to quantify the radioactivity picked up on the gamma probe and further to have a pre operative picture of the identified SLN. Injection of ^99 m^TcSC around the primary tumor site is performed at least 2 hours before or even the afternoon before surgery the next morning. The injection is reported to be painful as local anesthesia in the vicinity should be avoided. Another advantage is the possibility to perform a scintigraphy beforehand to identify and localize the SLN before surgery. This may be of great help where the SLN isolated in an unexpected area.

One of the main disadvantages is the often close proximity of the primary tumor to the first potential sentinel node where identification may be difficult [25]. This is especially true in mouth floor tumors and may lead to difficult or an inability to differentiate between the primary tumor and sentinel node.

Use of ICG in sentinel node identification for oropharyngeal cancer has to the authors knowledge not been published before. There are significant potential benefits in this new method.

The method is very simple and except for the equipment investment relatively cheap to run due to the relative small cost of the ICG compound. No pre planning needed as with conventional Technesium 99 labelled sulfur colloid and one can start with the sentinel node identification within seconds after injection. Few adverse reactions to ICG have been reported, however care should be taken in patients sensitive to iodide.

Clear visualization is possible if 5 mm or less of tissue covers the lymph node. Lymph pathways may be visualized that may lead to deeper lying draining lymph nodes. Uptake of ICG by other tissues like the submandibular gland was noted, but easily distinguished as non lymphatic tissue. An assistant can hold the infrared video camera and an area can be explored in real time.

Potential disadvantages are the lack of a pre operative visualization of potentially affected lymph nodes as with lymphoscyntography that may lead to larger incisions to gain access to the affected area, the inability to visualize the affected sentinel node in the presence of more than 5 mm of soft tissue coverage as well as a learning curve in the use of the infra red video camera and its settings. Furthermore no numerical or other quantification exists to be able to compare different areas of uptake. Green discoloration of the injected area did not have a marked influence on the resection of the primary tumor, however when unclear mucosal disease exists it is suggested to mark the mucosal resection borders before injection of the ICG is done.

## Conclusion

There are shortcomings in the current traditional ways of sentinel lymph node detection. Sentinel lymph node mapping by ICG fluorescence in patients with oropharyngeal cancer is a new potential method of demonstrating the first station lymph drainage basin as well as the associated lymph drainage patterns as it allows for direct visualization. Further development with controlled studies in this field is needed.

## Abbreviations

(ICG): Indocyanin Green; (SLN): Sentinel lymph node; (SNB): Sentinel Node Biopsy; (IHC): Immuno histochemistry.

## Competing interests

The authors declare that they have no competing interests.
